# Competence for Natural Transformation Is Common among Clinical Strains of Resistant *Acinetobacter* spp.

**DOI:** 10.3390/microorganisms7020030

**Published:** 2019-01-24

**Authors:** Sara Domingues, Natasha Rosário, Ângela Cândido, Daniela Neto, Kaare M. Nielsen, Gabriela J. Da Silva

**Affiliations:** 1 Faculty of Pharmacy, University of Coimbra, 3000-458 Coimbra, Portugal; narosario@live.com.pt (N.R.); angelandrade_93@hotmail.com (Â.C.); danielaneto471@hotmail.com (D.N.); gjsilva@ci.uc.pt (G.J.D.S.); 2 Centre for Neuroscience and Cell Biology, University of Coimbra, 3004-517 Coimbra, Portugal; 3 Department of Life Sciences and Health, Oslo Metropolitan University, N-0130 Oslo, Norway; kamni@oslomet.no

**Keywords:** *Acinetobacter baumannii*, *Acinetobacter nosocomialis*, clinical isolates, horizontal gene transfer, natural transformation, competence, osmolarity

## Abstract

Horizontal gene transfer events provide the basis for extensive dissemination of antimicrobial resistance traits between bacterial populations. Conjugation is considered to be the most frequent mechanism behind new resistance acquisitions in clinical pathogens but does not fully explain the resistance patterns seen in some bacterial genera. Gene transfer by natural transformation has been described for numerous clinical isolates, including some *Acinetobacter* species. The main aim of this study was to determine to what extent clinical, resistant *Acinetobacter* spp. isolates, express competence for natural transformation. Twenty-two clinical *Acinetobacter* spp. isolates collected over a 16-year time period, from five different geographical separated and/or distinct Portuguese Hospitals were tested for natural transformability. Fourteen isolates, including 11 *A. baumannii*, 2 *A. nosocomialis* and 1 *Acinetobacter* sp., were identified as competent on semisolid media facilitating surface-motility. Competent *Acinetobacter* isolates were found in all the hospitals tested. Furthermore, osmolarity was shown to influence the uptake of exogenous DNA by competent *A. baumannii* A118. Our study demonstrates that natural competence is common among clinical isolates of *Acinetobacter* spp., and hence likely an important trait for resistance acquisition.

## 1. Introduction

Antimicrobial resistance is considered a major threat to public health [1]. Most pathogenic microorganisms have acquired some resistance to many of the commonly used antibiotics. Among these are the so-called ESKAPE pathogens, which include *Enterococcus faecium*, *Staphylococcus aureus*, *Klebsiella pneumoniae*, *Acinetobacter baumannii*, *Pseudomonas aeruginosa* and *Enterobacter* spp. [2]. These are highly important nosocomial pathogens that are now becoming resistant to the majority of the available antibiotics. *A. baumannii* strains were initially susceptible to most of the antimicrobials in the early 70s, when resistance started to develop [3]. *A. baumannii* has emerged as an important nosocomial pathogen, and nowadays some strains are only susceptible to carbapenems and colistin [4]. Due to the lack of therapeutic options, this species has been classified as critical in the priority pathogens list released by the World Health Organization in 2017 [5].

Horizontal gene transfer (HGT) mechanisms play a crucial role in the dissemination of antimicrobial resistance worldwide [6]. Conjugation is often reported as the causal mechanism of gene transfer associated with the wide dissemination of many resistance genes [1]. However, resistance in *A. baumannii* cannot be fully explained by the acquisition of conjugative plasmids [4]. It is well known that environmental *Acinetobacter* species can express competence and undergo natural transformation [7,8]. Competence development contributes to the acquisition of antimicrobial resistance genes and mobile genetic elements from unrelated species in *Acinetobacter* [9]. However, this HGT mechanism has been poorly explored in clinical strains, which are often multidrug resistant. In 2010, an *A. baumannii* clinical isolate with the ability to undergo natural transformation was identified for the first time in Buenos Aires [10]. Later, an *Acinetobacter nosocomialis* strain, another important hospital pathogen isolated in the United States, was also reported to be naturally transformable [11,12]. Three other studies also described the presence of naturally competent *A. baumannii* isolates of human clinical [13], animal clinical [14] and animal non-clinical [15] origin. Transformability of *A. baumannii* has been shown to be influenced by different conditions such as the presence of Ca^2+^-ions and albumin [14,16], the pH [14,16], the presence of sub-inhibitory concentrations of mitomycin C, nalidixic acid and meropenem [17], or the agarose concentration [14]; however, the influence of these parameters seems to be strain-dependent [14,16]. Despite the fact that natural transformation is now well established as a common trait in *Acinetobacter* species, only one study has screened the ability to undergo natural competence in a set of human clinical isolates [13]. The primary objective of this work was to determine to what extent human clinical isolates of *Acinetobacter* species associated with human infections are able to express competence during growth in vitro. The effects of osmolarity variations in competence and in cells growth were also tested.

## 2. Materials and Methods

### 2.1. Bacterial Isolates and Growth Conditions

From our collection of clinical *Acinetobacter* spp., we selected and tested 22 kanamycin susceptible clinical isolates collected between 1992 and 2008. The isolates originated from five different geographical and/or distinct Portuguese hospitals (Table 1) and had different resistant profiles. The naturally competent *A. baumannii* A118 clinical strain was included as a positive control for transformability [10]. 

Bacteria were grown in Luria-Bertani (LB) (Fluka, Saint Louis, MO, USA) agar (Liofilchem, Roseto d. Abruzzi, Italy) medium and incubated at 37 °C. The ability of the various isolates to undergo natural transformation was examined during growth on plates containing 0.5% agar (Liofilchem), 5 g/L tryptone (Difco, Sparks, MD, USA) and 2.5 g/L sodium chloride (Scharlau, Sentmenat, Spain) [13], here called Motility Medium (MM).

Transformant cells were selected on LB plates supplemented with 30 µg/mL of kanamycin (Sigma, Saint Louis, MO, USA). 

The competent isolates detected in this study were identified to the species level by sequencing of the *rpoB* gene [18] (sequencing provider STAB VIDA, Portugal).

### 2.2. Growth Curve Under Different Sodium Chloride Concentrations

An overnight culture of *A. baumannii* A118 was prepared by inoculation of a single colony into 5 mL of LB broth, followed by incubation at 37 °C with good aeration (120 rpm). A 1:100 dilution into 50 mL of fresh LB broth containing tryptone (10 g/L), yeast extract (5 g/L) (Difco) and sodium chloride (0.5, 2.5, 5, 7.5 or 10 g/L) was done, followed by incubation at 37 °C and agitation at 120 rpm for 320 min. Samples were collected at 30 and 60 min and then each 20 min, and the optical density at 600 nm (OD_600nm_) was measured in a spectrophotometer (Genesys 10UV, Thermo Scientific, Madison, WI, USA). The growth curves were constructed plotting the OD_600nm_ values versus the incubation time.

### 2.3. Natural Transformation

Natural competence of the 22 clinical *Acinetobacter* spp. isolates was tested with the transformation protocol described by Wilharm et al. [13]. Briefly, a single colony was suspended in 20 µL of sterile phosphate-buffered saline (PBS) and mixed with 20 µL of DNA (4 µg in water); the MM was stabbed seven times depositing 2 µL of the transformation mixture each time; the plate was sealed with Parafilm and incubated at 37 °C for 24 h; bacteria were recovered from the medium surface and suspended in 1 mL of PBS, followed by selection on LB agar with kanamycin. Initial transformation of *A. baumannii* 121 was performed with purified chromosomal DNA from the *A. baumannii* mutant 179, which has a kanamycin resistance gene inserted into the sulphite reductase gene [13], kindly provided by G. Wilharm. DNA extracted from a transformant named 121-1, containing the kanamycin marker, was then used as transforming DNA for all subsequent transformation tests. Detection of competent isolates relied on the incorporation of the marker into the chromosome of the recipient cell by homologous recombination. Negative controls were always included in each transformation experiment, using water instead of donor DNA. Three independent transformation experiments were performed for all isolates that initially failed to produce transformants.

The same transformation protocol was used in transformation assays with the competent *A. baumannii* A118 under different concentrations of sodium chloride (0.5, 2.5, 5, 7.5 or 10 g/L). 

The transformation frequency was calculated as the ratio between the number of transformants and the total number of cells. A minimum of two independent transformation experiments, each repeated in triplicate, were done.

### 2.4. Transformants Confirmation

The Colony Forming Units (CFUs) that grew on LB plates with kanamycin were considered possible transformants. Selected cells were re-streaked and tested by PCR targeting the sulphite reductase gene [13], using the MasterMix Dynazyme II (Finnzymes, Finland). Bacterial cells that acquired the transforming DNA produce a PCR amplicon of approx. 2400 bp, while in wildtype cells this locus gives an amplicon size of 1200 bp due to the absence of the insert (G. Wilharm, personal communication). 

## 3. Results and Discussion

Fourteen clinical *Acinetobacter* spp. isolates were detected as naturally transformable (Table 1). The majority were identified as *A. baumannii* (*n* = 11), the most relevant nosocomial species, while two were *A. nosocomialis* and one isolate could not be identified to the species level. The ability to undergo natural transformation was detected in isolates from all hospitals, which suggests that resistant isolates with the ability to develop competence are widespread in the clinical environment. Moreover, such strains with the ability to express competence have been circulating in the hospital environment for a long time. This HGT trait can explain the increased antimicrobial resistance seen in this species in the last decades. The acquisition events of antimicrobial resistance by natural transformation may have been further promoted by the selection exerted by the increased use of antibiotics in the hospital environment. A recent study observed that strains circulating in the hospital environment show evidence of HGT and other genetic events, making almost every *A. baumannii* strain unique, despite their genetic relatedness determined by conventional typing methods such as MLST [19]. 

Although the donor DNA used in this study was homologous to *A. baumannii* (gene A1S_2846 in *A. baumannii* ATCC 17978) [13], two *A. nosocomialis* isolates were found to be transformable. Both species belong to the *Acinetobacter calcoaceticus-A. baumannii* complex that share genetic homology and are frequently found in the clinical environment [20]. Increasing genetic divergence reduces transformation frequency in *Acinetobacter* [21]. We can therefore not exclude the possibility that some of the other *Acinetobacter* spp. isolates tested here might be transformable but are so genetically divergent from the *A. baumannii* donor DNA that they turned out negative. 

**Figure 1:**
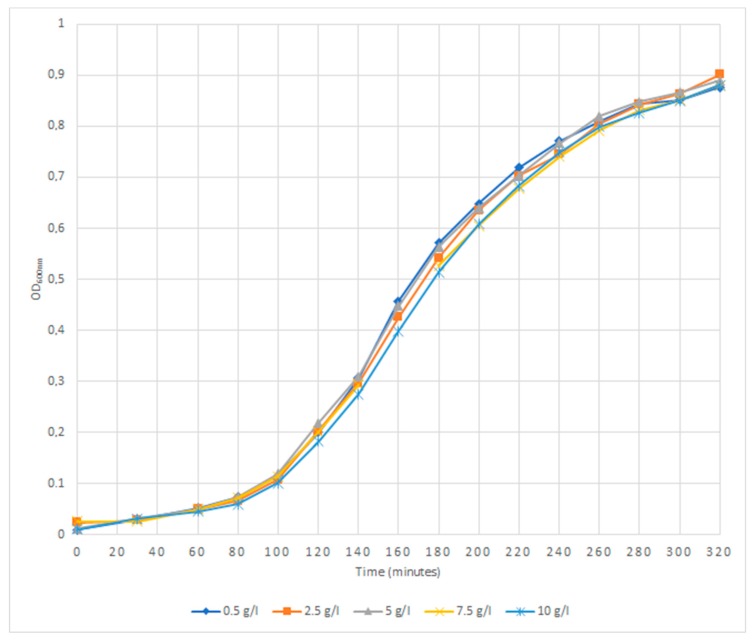
Effect of sodium chloride on the growth of *Acinetobacter baumannii* A118.

Several factors influence the natural transformation of *A. baumannii* cells. Two studies observed differences in the natural transformability of *A. baumannii* isolates depending on the type of transforming DNA (chromosomal versus plasmid DNA) [13,14]. However, a recent study showed that the transformability of *A. baumannii* A118 was not affected by the donor DNA type, temperature and osmolarity; on the other hand, transformation was influenced by the pH of the medium, with a higher transformation frequency obtained at 7.5, and both the presence of Ca^2+^-ions and albumin were shown to induce competence [16]. The authors concluded that the transformation frequency is impacted by the medium´s composition. The influence of different parameters seems however to be strain dependent, as in a recent study with *A. baumannii* AB5075, albumin was shown to decrease transformability, and the optimal pH for transformation was between 5.3 and 6.1; this study also showed that transformation frequencies increase with agarose concentration [14]. In our study, we used a different medium, MM, as well as different conditions for DNA exposure. In our experimental conditions, we observed that osmolarity influenced the transformability of *A. baumannii* A118, with transformation rates occurring at similar levels (10^−5^) in medium with 0.5, 2.5 and 5 g/L of sodium chloride, but two orders of magnitude lower (10^−7^) under higher amounts of salt (Table 2). The transformability was not related to differences in the growth rate of the cells, as growth was similar in all tested concentrations (Figure 1). In addition, we observed that transformation frequencies in semisolid medium were higher (10^−5^) than those obtained in liquid medium in other studies (10^−7^) [16]. Therefore, in addition to the specific composition of the growth medium, the overall growth conditions and pattern of DNA exposure also influence the transformability of *A. baumannii* A118. This finding suggests that transformability will change with environments and the various opportunities for growth of *A. baumannii* cells in vivo.

The previous reports and the present findings confirm that resistant clinical bacteria species, *A. baumannii* and *A. nosocomialis* are able to undergo natural transformation, supporting the hypothesis that natural transformation might be a mechanism for resistance acquisition in the clinical setting. Components of some biological fluids (Ca^2+^-ions and albumin) can induce transformation in *A. baumannii,* suggesting that *Acinetobacter* can develop competence in vivo [16]. A recent study has shown higher transformability of two *A. baumannii* clinical strains in biologic fluids containing a significant amount of albumin, such as pleural fluid, ascites fluid and whole blood [22]. Here, we also show that despite the frequency differences seen under the various concentrations of sodium chloride, transformation of *A. baumannii* A118 occurs at detectable levels in all tested salinity conditions, including those found in vivo (usually Na^+^ is present at a 3 g/L in human serum) (Table 2). 

Some of the *A. baumannii* isolates did not produce transformants in all replicates (data not shown). This might indicate that competence is tightly regulated in the studied bacterial population and that small variations in growth conditions led to minor differences in growth trajectories and conditions that altered their level of competence. Transformation can also be strain dependent; more strains need to be tested in order to widen our understanding of the true distribution of competence among *Acinetobacter* spp. More detailed system studies should be performed to fully dissect competence triggering conditions. Knowledge of the environmental conditions and bacterial population features that induce and maintain competence in clinical isolates is crucial to fully understand bacterial adaptation, particularly for antimicrobial agents, one of the biggest problems of this century.

## Figures and Tables

**Table 1:** Natural competence in clinical *Acinetobacter* spp. isolates.

Isolate	Species	Year of Isolation	Hospital/Region	Natural Competence ^a^
013	*A. nosocomialis*	1992	HUC/Coimbra	+
015	*A. baumannii*	1994	HUC/Coimbra	+
113	*A. baumannii*	1998	HUC/Coimbra	+
118	*A. bereziniae*	1998	HUC/Coimbra	-
121	*A. baumannii*	1998	HUC/Coimbra	+
129	*A. baumannii*	1998	HUC/Coimbra	+
132	*A. baumannii*	1998	HUC/Coimbra	+
138022 F1Ev	n.d.	2008	HES/Évora	-
144417 M1Ev	*A. baumannii*	2008	HES/Évora	+
213	n.d.	2004	HSAC/Lisbon	-
241	*A. baumannii*	2005	HSAC/Lisbon	+
245	*A. baumannii*	2005	HSAC/Lisbon	-
274	*A. baumannii*	2006	HSAC/Lisbon	-
292	*A. baumannii*	2006	HSAC/Lisbon	+
319	*A. baumannii*	2007	HSAC/Lisbon	+
326744 C1Ev	*A. nosocomialis*	2008	HES/Évora	+
3605	n.d.	1994	Porto	-
3625	*A. baumannii*	1995	Porto	+
532331 A1Ev	*A. baumannii*	2008	HES/Évora	+
545663 F2Ev	*A. baumannii*	2008	HES/Évora	-
065	*Acinetobacter* sp.	1999	HSM/Lisbon	+
65FFC	*A. baumannii*	1998	HUC/Coimbra	-

^a^ Detected by growth of transformant cells in LB with kanamycin 30 µg/ml and confirmed by PCR. + competence detected; - competence not detected. n.d.: not determined. HES: Espírito Santo Hospital; HSAC: Santo António dos Capuchos Hospital; HSM: Santa Maria Hospital; HUC: University Hospitals of Coimbra.

**Table 2:** Effects of sodium chloride on the transformation frequency of *Acinetobacter baumannii* A118 in semi-solid medium.

NaCl Concentration	Mean Number of Transformants (CFU) ± SD	Mean Number of Total Cells (CFU) ± SD	Transformation Frequency
0.5 g/L	(4.73 ± 3.11) × 10^4^	(1.14 ± 0.65) × 10^8^	4.1 × 10^−5^
2.5 g/L	(3.65 ± 4.30) × 10^4^	(6.38 ± 3.30) × 10^8^	5.7 × 10^−5^
5 g/L	(9.11 ± 6.68) × 10^3^	(5.27 ± 3.04) × 10^8^	1.7 × 10^−5^
7.5 g/L	(5.28 ± 56.1) × 10^2^	(8.14 ± 8.61) × 10^8^	6.5 × 10^−7^
10 g/L	(1.35 ± 1.58) × 10^2^	(7.88 ± 2.32) × 10^8^	1.7 × 10^−7^
